# Core outcome set development: the effect of Delphi panel composition and feedback on prioritisation of outcomes

**DOI:** 10.1186/1745-6215-14-S1-P77

**Published:** 2013-11-29

**Authors:** Rhiannon Macefield, Natalie Blencowe, Sara Brookes, Marc Jacobs, Mirjam Sprangers, Paula Williamson, Jane Blazeby

**Affiliations:** 1 University of Bristol, Bristol, UK; 2 Academic Medical Centre/University of Amsterdam, Amsterdam, The Netherlands; 3 University of Liverpool, Liverpool, UK; 4 University Hospitals Bristol NHS Foundation Trust, Bristol, UK

## 

Delphi methods are common in the development of core sets with panels including patients and healthcare professionals (HCPs) as important key stakeholders. Individuals are provided with feedback between survey rounds but methods for considering different stakeholders' views are lacking. This study explored the influence of randomised stakeholder feedback on responses.

In a survey to develop a core set for oesophageal cancer surgery, 185 patients and 126 HCPs rated 67 items on a scale of 1: not essential to 9: absolutely essential (Round 1). Feedback on each item was provided in Round 2 with participants randomly allocated to 1) patients receiving own-group feedback, 2) patients receiving both patient and HCP feedback, 3) HCPs receiving both patient and HCP feedback and 4) HCPs receiving own-group feedback. "Important" items were defined using a predetermined cut-off (rated 7-9 by >70% of participants). Ratings were summarised as mean scores and differences between randomised groups compared.

147 patients and 107 HCPs completed Round 2. Patients receiving only own-group feedback rated more items as important (Group 1: 20 important items versus Group 2: 18 important items). However, HCPs receiving both-group feedback rated more items as important (Group 3: 21 important items versus Group 4: 16 important items). For individual items, the difference in mean scores between randomised groups was small, although there was a trend for HCPs ratings to be influenced by patient feedback.

Evidence suggests panel composition and the feedback provided may influence results. Researchers should carefully consider these methodological factors when designing a Delphi study.

